# Established Time Goals Can Increase the Efficiency of Trauma Resuscitation

**DOI:** 10.7759/cureus.9524

**Published:** 2020-08-02

**Authors:** Mark A Taylor, Hilary A Hewes, Carol D Bolinger, Stephen J Fenton, Katie W Russell

**Affiliations:** 1 Department of Surgery, University of Utah Health, Salt Lake City, USA; 2 Department of Emergency Medicine, Primary Children's Hospital, Salt Lake City, USA; 3 Department of Pediatric Surgery, Primary Children's Hospital, Salt Lake City, USA

**Keywords:** video review, quality improvement, injured child

## Abstract

Introduction

Our institution uses video review as a quality improvement tool. Starting in March 2018, we specifically focused on meeting certain time goals during trauma resuscitation aimed at decreasing time to final disposition. The purpose of this study was to evaluate the effect of establishing strict time goals on total time spent in the trauma bay by pediatric trauma patients.

Materials and methods

A retrospective review of all level I trauma activations at a level I pediatric trauma center between November 2017 and December 2018 was performed via manual review of the recorded trauma activations. Data on key time points such as time from arrival to transfer to gurney, to completion of primary survey, to chest x-ray, to Emergency Medical Services (EMS) report, to CT scan, and to disposition (CT or admission/operating room [OR] if no CT scan was performed) were analyzed and compared between the cohort of patients prior to implementation of the time goals with that after. The cohort of patients who presented between March 2018 and May 2018 were excluded to allow for time for the intervention to take effect.

Results

There were 13 level I trauma activations before implementation of the time goals and 41 after. There was a significant decrease in time to transfer to gurney (1.8 minutes vs. 1.0 minutes; p=0.02), to CT scan (18.8 minutes vs. 14.2 minutes; p=0.01), and to disposition (18.3 minutes vs. 14.9 minutes; p=0.047). There was no decrease in time to completion of primary survey, EMS report, or chest x-ray.

Conclusions

Utilizing video review in pediatric trauma as a quality improvement initiative with a focus on meeting specific time goals for key elements of the activation led to decreased total time in our trauma bay with critically ill patients.

## Introduction

Video review in trauma was first described in 1988 by Hoyt et al. [1]. A significant portion of trauma programs in the US utilize a trauma video review system, with those not using video review most frequently citing legal or privacy issues as the primary concern with implementation [2]. While medical team participants in video review report provider anxiety as the main adverse effect of conducting video review, the vast majority of participants feel that it provides significant educational value [3]. Video review in trauma has been shown to improve trauma team function, identify and decrease errors during trauma resuscitation, and increase compliance to Advanced Trauma Life Support (ATLS) protocols [4-6].

Our level I pediatric trauma center instituted a trauma video review system in 2015. In watching videos of traumas, we noticed that there was a significant amount of idle time throughout a trauma activation. Given data suggesting that decreasing duration of a trauma resuscitation can improve outcomes, in March 2018, we introduced a quality improvement initiative aimed at meeting certain time goals during the trauma resuscitation [7]. Reviewing resuscitations and focusing on these time goals in video review allowed for the identification of delays in care and fostered discussion on how these delays could be mitigated. Although excess time has been shown to increase mortality in severely injured trauma patients, there is limited data on the effect of video review focusing on meeting specific time goals on total time spent in the trauma bay prior to final disposition [7]. The aim of this study was to evaluate the effect of creating specific time goals for key steps in a trauma activation in order to reduce the total time spent in trauma bay resuscitation of a severely injured pediatric patient prior to final disposition.

## Materials and methods

A retrospective review of all level I trauma activations that presented to our level I pediatric trauma center between November 2017 and December 2018 was performed. The cohort of patients who presented prior to institution of the specific time goals (November 2017 to February 2018) was compared with the cohort of patients who presented after (June 2018 to December 2018). Patients who presented within the three months after institution of time goals (March 2018 to May 2018) were excluded from analysis to allow for time for the intervention to take effect. The time goals were as follows: <3 minutes from patient arrival to transfer to gurney, <5 minutes to completion of primary survey, <7 minutes to completion of Emergency Medical Services (EMS) report and chest x-ray, <15 minutes to CT scan, and <1 hour to admission/operating room (OR). Prior to this intervention, the trauma surgeons, emergency department (ED) staff physicians, fellows, and residents, ED trauma nurses, and trauma advanced practice providers met for a monthly video review of trauma resuscitations as a means of learning from prior trauma patients to improve the process of trauma resuscitation. After the intervention, these meetings continued, but with a focus on the above time goals. As not all members of a trauma team could be present at these video review sessions, information about these time goals and areas in which a trauma team could improve were disseminated via email, nursing meetings, resident education, and the trauma process improvement and patient safety meetings.

Demographic data including age and gender were collected and summarized by reporting the mean for age and percentages for gender. Data on time from arrival of patient into the bay to transfer to gurney, to completion of primary survey, to chest x-ray, to EMS report, to CT scan, and to disposition (CT or admission/OR if no CT scan was performed) were analyzed and compared between the two cohorts. Elapsed time to these endpoints was determined based on manual review of the trauma videos. Time data were summarized by reporting mean and standard deviation (SD) for all variables. For statistical analysis, a t-test was used to compare mean time to the specified endpoints between the two cohorts. Statistical significance was assessed at the 0.05 level using two-tailed tests.

## Results

There were 13 level I trauma activations before implementation of specific time goals and 41 after. The average age of patients was similar between the two cohorts (8.9 years [SD 5.3] vs. 9.7 years [SD 5.5]; p=0.34). Overall, the patients were 63% male and 37% female, and there was no statistical difference in gender between the two cohorts. There was no difference between the before implementation and after implementation cohorts in the percentage of patients who received a chest x-ray (84% vs. 78%, p=0.53) or CT scan (92% vs. 78%, p=0.45).

The average time to gurney (1.8 minutes vs. 1.0 minutes; p=0.02), average time to CT scan (18.8 minutes vs. 14.2 minutes; p=0.01), and average time to disposition (18.3 minutes vs. 14.9 minutes; p=0.047) were all significantly decreased in the post-intervention group (Table 1). The average time to completion of primary survey, EMS report, and chest x-ray were all similar between the two cohorts.

**Table 1:** Comparison of times in the trauma bay before and after implementation of video review focusing on specific time goals SD: standard deviation; EMS: Emergency Medical Services

Average time, in minutes, from arrival to… (SD)	Before intervention	After intervention	P-value
	n=13	n=41	
Transfer to gurney	1.8 (0.6)	1.0 (0.5)	0.02
Completion of primary survey	1.7 (0.9)	1.8 (0.7)	0.75
EMS report	4.6 (1.5)	4.1 (2.0)	0.36
Chest x-ray	4.9 (1.9)	4.8 (3.3)	0.91
CT scan	18.8 (4.3)	14.2 (5.5)	0.01
Disposition	18.3 (4.5)	14.9 (5.4)	0.047

## Discussion

Our study demonstrates that using video review in pediatric trauma resuscitations as a quality improvement initiative with specific time goals for key elements of the activation led to decreased total time in our trauma bay with critically ill patients. We demonstrated a 44% and 24% decrease in time from arrival to transfer to gurney and to CT scan, respectively. Furthermore, institution of these time goals led to a 3.4-minute decrease in total time spent in the trauma bay. While we did not find a significant decrease in time from arrival to completion of primary survey, EMS report, or chest x-ray, these goals were being met prior to the intervention suggesting there was already little inefficiency in the process of performing these tasks.

Since the Institute of Medicine released *To Err Is Human: Building a Safer Health System*, a report issued in 1999 that identified healthcare systems issues as a major source of patient morbidity and mortality, improving the quality of healthcare has become a major goal [8,9]. In trauma, excessive time in the trauma bay has been shown to negatively affect outcomes in severely injured trauma patients [7]. There are many inefficiencies that occur during the course of a trauma activation that contribute to prolonged times in the trauma bay [4,5]. Furthermore, there is significant data that shows, both objectively and subjectively, that video review can improve the quality of trauma resuscitations [4-6]. While our study was not designed to look at patient level outcomes, the decrease in time spent in the trauma bay likely lead to improved patient care by streamlining the trauma resuscitation.

With an attempt to improve the quality of care provided to pediatric trauma patients at our institution using the Plan-Do-Study-Act (PDSA) method of quality improvement, we identified an area for improvement during video review: times to accomplish key elements of a trauma resuscitation were not efficient [8]. We, therefore, defined strict time goals for these processes, and then studied the effect of focusing on these time goals during activations. Future efforts in quality improvement will focus on identifying the factors in the trauma process that lead to this decrease in time in the trauma bay through further PDSA cycles.

This study is limited because of its single-institution, retrospective design. Trauma systems across the country are run very differently, and it is not clear whether this study is generalizable across systems. Multi-institutional studies may give a better understanding of the utility of video review in various systems. Furthermore, we did not specifically investigate what changed in the process of running trauma activations that lead to the decrease in time. While the Hawthorne effect may have played a role in decreasing time spent in the trauma bay, future PDSA cycles will be performed to identify what behaviors actually changed to cause this effect, and to investigate how these behaviors can be further optimized to improve the efficiency of trauma activations. Finally, we did not investigate patient outcome measures in this study, and, thus, cannot say for certain that this decrease in time spent during trauma resuscitation lead to improved outcomes.

## Conclusions

Utilizing video review in pediatric trauma as a quality improvement initiative with a focus on specific time goals for key elements of the activation led to decreased total time in our trauma bay with critically ill patients. Inclusion of all members of the trauma team in this quality improvement initiative is vital to meeting these time goals. Specific inefficiencies in a trauma resuscitation will be identified and improved using further quality improvement cycles.

## References

[1] Hoyt DB, Shackford SR, Fridland PH, Mackersie RC, Hansbrough JF, Wachtel TL, Fortune JB (1988). Video recording trauma resuscitations: an effective teaching technique. J Trauma.

[2] Rogers SC, Dudley NC, McDonnell W, Scaife E, Morris S, Nelson D (2010). Lights, camera, action… spotlight on trauma video review: an underutilized means of quality improvement and education. Pediatr Emerg Care.

[3] Davis L, Johnson L, Allen SR, Kim PK, Sims CA, Pascual JL, Holena DN (2013). Practitioner perceptions of trauma video Review. J Trauma Nurs.

[4] Oakley E, Stocker S, Staubli G, Young S (2006). Using video recording to identify management errors in pediatric trauma resuscitation. Pediatrics.

[5] Hamilton NA, Kieninger AN, Woodhouse J, Freeman BD, Murray D, Klingensmith ME (2012). Video review using a reliable evaluation metric improves team function in high-fidelity simulated trauma resuscitation. J Surg Educ.

[6] Wurster LA, Thakkar RK, Haley KJ (2017). Standardizing the initial resuscitation of the trauma patient with the Primary Assessment Completion Tool using video review. J Trauma Acute Care Surg.

[7] Meizoso JP, Ray JJ, Karcutskie CA (2016). Effect of time to operation on mortality for hypotensive patients with gunshot wounds to the torso: the golden 10 minutes. J Trauma Acute Care Surg.

[8] Minami CA, Sheils CR, Bilimoria KY (2016). Process improvement in surgery. Curr Probl Surg.

[9] Institute of Medicine; Committee on Quality of Health Care in America (2000). To Err is Human: Building a Safer Health System.

